# Ubiquitin-Like Modifier Activating Enzyme 1 as a Novel Diagnostic and Prognostic Indicator That Correlates With Ferroptosis and the Malignant Phenotypes of Liver Cancer Cells

**DOI:** 10.3389/fonc.2020.592413

**Published:** 2020-12-03

**Authors:** Yiru Shan, Guang Yang, Haixia Huang, Yehan Zhou, Xiangyu Hu, Qiuhong Lu, Peng Guo, Jun Hou, Li Cao, Fuhua Tian, Qi Pan

**Affiliations:** ^1^Department of Oncology, Jiulongpo People’s Hospital of Chongqing, Chongqing, China; ^2^Department of Urology Surgery, The First Affiliated Hospital of Chongqing Medical University, Chongqing, China; ^3^Department of Critical Care Medicine, Children’s Hospital of Chongqing Medical University, Ministry of Education Key Laboratory of Child Development and Disorders, China international Science and Technology Cooperation Base of Child Development and Critical Disorders, Chongqing, China; ^4^Department of Pathology, Sichuan Cancer Hospital & Institute, Sichuan Cancer Center, School of Medicine, University of Electronic Science and Technology of China, Chengdu, China; ^5^Department of Dermatology, Chongqing Traditional Chinese Medicine Hospital, Chongqing, China; ^6^Department of Orthopaedics, Jiulongpo People’s Hospital of Chongqing, Chongqing, China; ^7^Department of Patient Service Center, Jiulongpo People’s Hospital of Chongqing, Chongqing, China; ^8^College of Bioengineering, “111 Project” Laboratory of Biomechanics & Tissue Repair Engineering, Key Laboratory of Biorheological Science and Technology, Ministry of Education, Chongqing University, Chongqing, China

**Keywords:** ferroptosis, hepatocellular carcinoma, *UBA1*, *Nrf2* signal transduction pathway, weighted gene coexpression network analysis

## Abstract

**Purpose:**

Ferroptosis is a type of cell death that is iron dependent, a characteristic that distinguishes it from necrosis, apoptosis, and autophagy. However, the ferroptotic mechanisms for hepatitis B virus-associated hepatocellular carcinoma (HCC) remain incompletely described.

**Methods:**

Two hepatitis B virus-associated HCC public datasets, GSE22058 (n=192) and GSE54238 (n=23), were obtained from the NCBI Gene Expression Omnibus (GEO) database. Bioinformatics methods, including weighted gene coexpression network analysis (WGCNA), Cox regression, and LASSO analysis, were used to identify signature markers for diagnosis and prognosis. CCK8, wound healing, Transwell migration/invasion, and ferroptosis assays were employed to explore the biological function of novel candidate markers weight gene coexpression network analysis.

**Results:**

In total, 926 differentially expressed genes (DEGs) were common between the GSE22058 and GSE54238 datasets. Following WGCNA, 515 DEGs derived from the MEturquoise gene module were employed to establish diagnosis and prognosis models in The Cancer Genome Atlas (TCGA) HCC RNA-Seq cohort (n=423). The score of the diagnostic model was strikingly upregulated in the TCGA HCC group (*p*<2.2e-16). The prognostic model exhibited high specificity and sensitivity in both training and validation (AUC=0.835 and 0.626, respectively), and the high-risk group showed dismal prognostic outcomes compared with the low-risk group (training: *p*=1.416e-10; validation: *p*=4.495e-02). Ubiquitin-like modifier activating enzyme 1 (*UBA1*) was identified among both diagnosis and prognosis signature genes, and its overexpression was associated with poor survival. We validated the expression level of *UBA1* in eight pairs of HCC patient tissues and liver cancer cell lines. *UBA1* silencing decreased proliferation, migration, and invasion in Huh7 cells while elevating the Fe^2+^ and malondialdehyde (MDA) levels. Additionally, these biological effects were recovered by oltipraz (an *Nrf2* activator). Furthermore, blocking *UBA1* strikingly repressed the protein expression levels of *Nrf2*, *HO-1*, *NQO1*, and *FTH1* in the *Nrf2* signal transduction pathway.

**Conclusion:**

Our findings demonstrated that *UBA1* participates in the development of HCC by modulating Huh7 phenotypes and ferroptosis *via* the *Nrf2* signal transduction pathway and might be a promising diagnostic and prognostic indicator for HCC.

## Introduction

Hepatocellular carcinoma (HCC) ranks fourth among the causes of cancer-related death worldwide (1), and hepatitis B virus (HBV) is the primary pathogenic factor (2). The prevalence of HCC among developing countries such as China shows an increasing trend (3, 4). Presently, no precise indicator is available for early disease diagnosis, contributing to the dismal HCC prognostic outcome. Thus, it is crucial to identify precise markers to improve HCC diagnosis and judge prognosis (5, 6).

Substantial progress has been made in microarray techniques regarding transcriptomics, which more comprehensively and precisely examines tumor transcription profiles. Applying bioinformatic analysis to microarray expression patterns can help to optimize possible precise markers. For example, some ubiquitin-activating enzymes related to human cancer occurrence have been identified (7, 8). Additionally, the abnormal expression of *UBA1*, an E1 enzyme for ubiquitin-activating enzymes, is associated with lung cancer (LC) (9) and cutaneous squamous cell carcinoma (SCC) (10), revealing the potential value of *UBA1* as an HCC diagnostic and prognostic marker.

Ferroptosis is a type of cell death that is iron dependent, a characteristic that distinguishes it from necrosis, apoptosis, and autophagy (11). Ferroptosis causes the accumulation of ferric iron while promoting lipid peroxidation. Inducing ferroptosis helps to selectively eliminate several cancer cells, revealing a novel strategy (12, 13). In recent years, some ferroptosis regulators have been found in some tumor cells. For example, activating the oxidative stress (OS) response gene *Nrf2* could avoid ferroptosis (12). Additionally, glutathione peroxidase-4 (*GPX4*), a typical selenium-dependent glutathione peroxidase distributed within mammals, plays an important role in suppressing the generation of lipid ROS in the process of ferroptosis (13). Nonetheless, the possible regulatory mechanism involved in ferroptosis is still unclear.

As suggested in this study, *UBA1* serves as a possible marker for diagnosis and prognosis prediction and regulates hepatocellular carcinoma (HCC) cell proliferation, invasion, migration, and ferroptosis through the *Nrf2* signal transduction pathway. Thus, integrating bioinformatic analysis with *in vitro* analysis allows a more effective contribution to HCC diagnosis and prognosis prediction.

## Materials and Methods

### HCC Patients

Human HCC tissue samples, together with corresponding noncarcinoma tissue samples, were obtained from HCC patients undergoing surgery at Sichuan Cancer Hospital. Sample collection was approved by the Research Ethics Committee of Sichuan Cancer Hospital. Each patient provided informed consent to participate in the study. All the collected samples were frozen immediately in liquid nitrogen until subsequent analysis.

### Collection of HCC Microarray Expression Patterns

To identify the differentially expressed genes (DEGs) between HCC tissue samples and normal liver tissue samples (NL), two published public datasets, GSE22058 (n=192) (14) and GSE54238 (n=23) (15), based on the GPL6793 and GPL16955 platforms, were utilized from the GEO database.

### DEG Identification

Following potent multichip mean background correction, the microarray matrix file was subjected to quantile normalization and expression calculation using Affymetrix to obtain the gene expression profiles. Additionally, Limma was used to construct the linear model together with Bayesian statistics. DEGs were identified from the HCC samples at the thresholds of *P <*0.05 and | Log2 (fold change, FC) |> 0.5.

### Weighted Gene Coexpression Network Analysis (WGCNA)

The DEGs between HCC and NL tissues were analyzed using the WGCNA R package (16). First, the DEG expression patterns, together with the corresponding clinical data, were downloaded. Next, the samples were clustered by Pearson’s correlation, and outliers were detected at the threshold of 10,000. Thereafter, Pearson’s correlation was applied to analyze each gene, and then a similarity matrix was established. Additionally, to identify the related modules, the soft-thresholding project was utilized in WGCNA to avoid screening the arbitrary cutoff. The β-value is a soft-thresholding parameter to emphasize the potent association of genes while penalizing the low associations, thus ensuring the scale-free network. Moreover, the gene network connectivity (k) was calculated by combining the gene adjacency with other genes to generate a network. The threshold power decision value was calculated according to the scale-free topological criterion, thereby mimicking the common network structure discovered in nature. In line with the scale-free topological criterion >0.8, β=16 was chosen in the present work. For the hierarchical clustering dendrograms, the adaptive branches were pruned using the cutreeDynamic function. Later, to better analyze the gene modules, the WGCNA package moduleEigengenes function was utilized to calculate the module eigengenes (ME) dissimilarity, regarded as the first principal component for a specific module as well as the typical gene expression pattern for a specific module. After selecting one cutoff line for the module dendrogram, all the modules were merged. Eventually, the adjacency was transformed into the topological overlap matrix (TOM), whereas the modules were subjected to hierarchical clustering analysis in accordance with the TOM-based dissimilarity measure, and the minimum size was 20.

### Selection of HCC Progression-Associated Hub Genes

The associations of coexpression modules with clinical conditions of HCC progression were examined. First, the gene significance (GS) was regarded as the log10 transformation of a specific *p*-value for the association of gene expression with the clinical condition. Second, principal component analysis was conducted to determine the module eigengenes (MEs), which were then chosen to represent the average measure for the integrated coexpression module. Finally, the correlation degrees of MEs with clinical characteristics were measured using Pearson’s correlation. Next, |moderate intensity correlation| >0.3 was used to identify significant modules at the *p*<0.05 threshold. The genes in those significant modules associated with significant metastatic characteristics were selected to identify critical genes at the thresholds of MM>0.8 and GS>0.2. To obtain the credible HCC progression-associated hub genes, the WGCNA package moduleTraitPvalue function was used to identify hub genes from the HCC progression-associated gene modules.

### Enrichment Analysis

GO annotation is a frequently adopted method to annotate genes and gene products and identify the characteristic biological performances for high-throughput transcriptome and genome patterns (17). Kyoto Encyclopedia of Genes and Genomes (KEGG, http://www.genome.jp) is commonly used in the systemic analysis of gene functions and associates genome data with functional data at a higher order (18). The DAVID database (https://david.ncifcrf.gov/) is usually used to map a specific user gene to correlated biological annotations and plays a critical role in successfully analyzing genes after high-throughput sequencing (19). In this study, DAVID was used to perform GO and KEGG pathway analyses to examine DEG functions. A difference of *p*<0.05 indicated statistical significance.

### Establishment of HCC Diagnosis and Prognosis Models

#### Diagnosis Model Analysis

The least absolute shrinkage and selection operator (LASSO) together with the random forest approach was used to identify significant genes to construct a signature for differentiating tumors from noncarcinoma samples. Finally, the genes identified by the two approaches overlapped, and the common ones were screened to building the diagnosis model according to logistic regression analysis (20).

#### Prognosis Model Analysis

Kaplan–Meier analysis was employed to calculate survival rates, and the log-rank test was utilized to determine the significance of differences among different survival curves. Univariate and multivariate analyses were performed based on the Cox proportional hazards model. After dimensionality reduction using the LASSO-Cox approach, the top genes related to HCC progression were chosen to construct the prognosis model by Cox regression analysis (21). The nomogram was constructed and validated following LASSO’s method (22). Next, receiver operating characteristic (ROC) curves were plotted, and the area under the ROC curve (AUC) values were calculated to analyze the model’s sensitivity and specificity (23).

### Immunohistochemical (IHC) Staining

HCC tissue samples were subjected to routine treatments, including dehydration, paraffin embedding, slicing into 4-μm sections, xylene deparaffinization, and gradient ethanol rehydration at ambient temperature. Sodium citrate was used to recover samples, 3% H_2_O_2_ was added to block the activity of endogenous peroxidase, and 5% bovine serum albumin (BSA) was added for 30 min of blocking at ambient temperature. Next, the primary anti‐*UBA1* antibody (No. 15912-1-AP; Proteintech, China) was incubated with the specimens at 4°C for 12 h. Next, horseradish peroxidase (HRP)‐conjugated secondary antibodies were incubated with the specimens for another 2 h at ambient temperature. Afterwards, the Cell and Tissue Staining HRP‐DAB kit (Beyond) was used for color development according to the manufacturer’s instructions, and an Orthophoto microscope (×40 and ×100) was used to capture images. We used image-pro_plus software to conduct semiquantitative analysis of the immunohistochemical results. The target and control regions were selected to measure the IOD value and area of the region, and the mean density (IOD/area) was calculated in two regions.

### Cell Culture and Transfection

The HCC HepG2 and Huh7 cell lines, as well as the immortalized noncarcinoma THLE-2 liver cell line, were purchased from the Cell Bank of the Chinese Academy of Sciences. The three cell lines were cultured in RPMI-1640 medium (Gibco, Grand Island, NY, USA) containing 10% fetal bovine serum (FBS; Gibco, Grand Island, NY, USA) and 1% penicillin-streptomycin and were incubated under 37°C and 5% CO_2_ conditions.

Three *UBA1*-siRNAs, along with the related negative control (NC) siRNAs, were provided by INVITROGEN (CA, US) to silence *UBA1* expression. In this study, the siRNA sequences were as follows: si-*UBA1* #1: ID s599; si-*UBA1* #2: 5′-GCGUGGAGAUCGCUAAGA-3′ (sense), 5′-UUCUUAGCGAUCUCCACG-3′ (antisense). However, the manufacturer did not provide the sequences for NC siRNAs. Next, the cells were plated in 6-well plates at a density of 1×10^5^ cells/well until 70%–80% cell confluence and then were harvested to conduct transient transfection. Subsequently, the cells were transfected with either siRNA or NC using Lipofectamine 2000 (Invitrogen, USA) to a final volume of 100 nM according to the manufacturer’s protocols. The target gene expression was then analyzed in cells transfected for 48 h.

### RNA Extraction and Reverse Transcription–Quantitative Polymerase Chain Reaction (RT–qPCR)

Total RNA was extracted from the cells using an RNAiso Plus kit (Takara, USA) according to the manufacturer’s protocol. Subsequently, the RNA was reverse transcribed into cDNA using the following temperature protocol: 37°C for 15 min, 85°C for 5 seconds, and 4°C for 5 min, using the PrimeScriptTM RT Reagent kit containing gDNA Eraser (Takara, USA). qPCR analysis was performed using SYBR Premix Ex Taq II (Takara, USA) and the CFX96 Touch Real‐Time PCR system (Bio‐Rad, USA) with the following thermocycling conditions: 95°C for 30 seconds, 40 cycles of 95°C for 5 seconds, and 60°C for 30 seconds. The relative gene expression data were quantified using the 2‐ΔΔCq method. *GAPDH* served as an internal control. The following primers were used: *GAPDH* forward, 5′‐CTTTGGTATCGTGGAAGGACTC‐3′ and reverse, 5′‐GTAGAGGCAGGGATGATGTTCT‐3′; *UBA1* forward, 5′‐CACAAGAGCAAGCTGATTGCAGGGAAGA‐3′ and reverse, 5′‐AAGGCAGGGCCAAGTTGAGGAAACCATT‐3′. All the reactions were performed in triplicate.

### Cell Proliferation Assay

The Cell Counting Kit-8 (CCK8, Dojindo, Japan) was adopted to analyze cell proliferation according to the manufacturer’s protocols. Briefly, cells were plated into 96-well plates at a density of 4×10^3^ cells/well. After culture for 0, 24, 48 and 72 h, the CCK-8 solution was added to the plates for 1.5 h of incubation in the dark. Next, the absorbance values were determined at 450 nm, and the number of viable cells was calculated.

### Wound-Healing Assay

A representative *in vitro* wound-healing assay was carried out to evaluate Huh7 cell migration ability with or without treatment. Briefly, cells were plated in 6-well plates until 100% cell confluence. Next, the cell surface was scratched using a sterile pipette tip, and cells were rinsed with PBS three times to remove the detached cells, followed by 24 h of culture within the serum-free medium under the same conditions. Finally, the images were acquired at 0 h and 24 h.

### Transwell Migration and Invasion Assays

Transwell assays were performed to examine Huh7 cell invasion and migration ability. First, Huh7 cells with or without treatment were suspended in serum-free medium. To test the cell migration ability, 100 µL of the cell suspension prepared above was collected and added to the upper chamber coated with Matrigel (BD Biosciences) in advance, whereas 600 µL of medium containing 10% FBS was loaded into the lower chamber. Next, the cells were plated into the upper chamber at a density of 4×10^4^, followed by 15 min of 4% paraformaldehyde fixation. Twenty-four hours later, the cells were removed from the chamber, and crystal violet (0.1%) was used to stain the treated cells for 10 min. Next, cells on the inner layer were carefully chosen, and 3 fields of view (FOV) were randomly selected from every sample to calculate the number of penetrating cells.

### Iron Assay

The iron assay kit (No. DIFE-250; BioAssay Systems, USA) was used to evaluate the relative iron content in cell lysates according to the manufacturer’s protocols.

### Lipid Peroxidation Assay

The relative level of malondialdehyde (MDA) in the cell lysate was assessed using a lipid peroxidation detection kit (No. A003-1-2; Nanjing Jiancheng College of Biotechnology, China) following the kit protocol.

### Western Blotting Assay

After isolating total protein from the cells, the BCA protein detection kit (Beyotime, Shanghai, China) was used to quantify the protein content. Next, 8% SDS-PAGE was applied to isolate proteins, which were later transferred onto PVDF membranes. Afterwards, 5% skimmed milk was used to block the membranes, which were incubated with primary antibodies overnight at 4°C. After washing with TBST three times, the membranes were incubated with secondary antibody for 1 h at room temperature, and TBST was used to wash the membranes three times. Finally, ECL solution (Wanleibio, Shenyang, China) was used for color development in the dark.

### Statistical Methods

All the values were presented as means ± standard deviation (SD). Statistical analysis was performed using GraphPad Prism analysis software. The *t-*test together with one-way ANOVA was adopted to assess the differences between two groups. Additionally, differences among three groups were examined using one-way ANOVA and LSD *t-*test. * *p*<0.05; ** *p*<0.01; # *p*<0.05; ## *p*<0.01.

## Results

### Identification of DEGs

**Figure 1:**
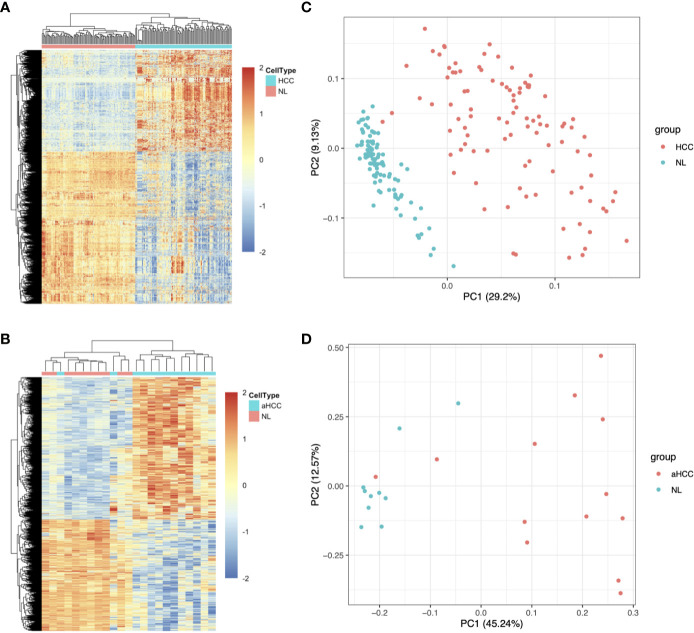
Upregulated and downregulated DEG distribution in HCC samples based on the GSE22058 and GSE54238 datasets. **(A, B)** DEG hierarchical clustering analysis. All the data input represents the log_2_ ratio of HCC intensity relative to the normal intensity. The red color represents markedly upregulated genes selected at *p <*0.05; the blue color represents markedly downregulated genes at *p <*0.05. **(C, D)** PCA was performed to separate HCC from nontumor samples. aHCC, advanced hepatocellular carcinoma.

Two hepatitis B virus-associated HCC public datasets, GSE22058 (n=192) and GSE54238 (n=23), were obtained from the GEO database. The Limma R package was used to analyze the expression patterns of DEGs acquired based on the GSE22058 (n=192) and GSE54238 (n=23) datasets (**Table 1**). In total, 3,004 DEGs were identified between 96 pairs of HCC and noncarcinoma samples in the GSE22058 dataset using the thresholds of | Log2 (FC) | > 0.5 and *p-*value < 0.05, among which 1,202 DEGs were upregulated and 1,802 DEGs were downregulated (**Figure 1A**). In the GSE54238 dataset, 4,553 DEGs were identified between 13 HCC and 10 noncarcinoma samples, among which 2,633 DEGs were upregulated and 1,920 DEGs were downregulated (**Figure 1B**). As suggested by principal component analysis (PCA), the DEGs obtained from the GSE22058 dataset helped to comprehensively distinguish HCC from noncarcinoma samples (**Figure 1C**). Such results suggested that the DEG expression profiles represented a typical feature to distinguish HCC from noncarcinoma samples. Similar findings were obtained from the GSE54238 dataset following PCA (**Figure 1D**).

**Table 1:** The acquisition of HCC microarray expression profiles.

Accession	Platform	Disease category	Sample size (case/control)	Country	Year
GSE22058	GPL6793	HCC	96/96	USA	2010
GSE54238	GPL16955	HCC	13/10	USA	2014

### Determination of the HCC Advance-Related Module by WGCNA

**Figure 2:**
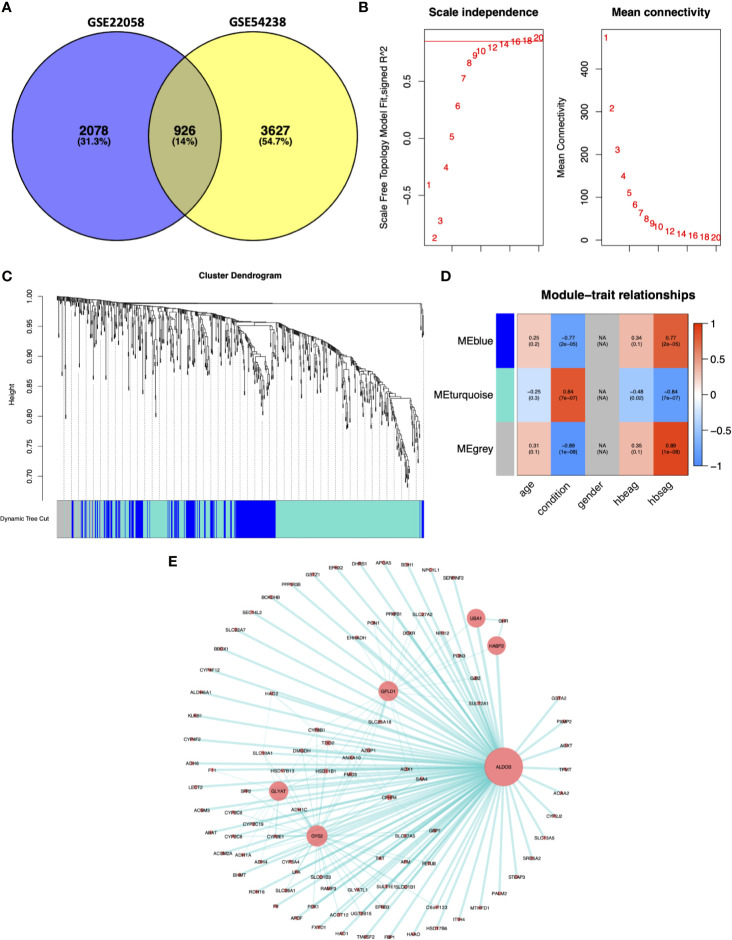
Weighted gene coexpression network analysis. **(A)** Common DEGs between the GSE22058 and GSE54238 datasets are displayed. **(B)** Soft-thresholding for the adjacency matrix. **(C)** Hierarchical clustering-derived gene dendrogram. The dendrogram shows average linkage hierarchical clustering of DEGs. The colors under the dendrogram indicate the diverse modules. **(D)** Associations of modules with clinical characteristics. Modules denoted *via* diverse colors are presented along the vertical axis. Correlation coefficients were expressed as numbers at related positions, whereas *p*-values were presented in brackets with the coefficients. **(E)** Network visualization for the MEturquoise module. The node size was proportional to the connectivity degree. HBeAg, hepatitis B e-antigen; HBsAg, surface antigen of hepatitis B virus.

We selected 926 DEGs that were common between the GSE22058 and GSE54238 datasets for further analysis (**Figure 2A**). The WGCNA algorithm was conducted to identify gene coexpression modules from the 926 DEGs. The soft-threshold was established through the softConnectivity function of WGCNA. When the power achieves 16, the scale-free topology fit index was up to 0.8. Therefore, we picked out power value 16 to evaluate 926 DEGs adjacency matrix and to build a coexpression module to determine the impact of soft-thresholding power on the scale-free fit index and the mean connectivity (**Figure 2B**). A hierarchical cluster analysis for 926 DEGs was performed to discover coexpression clusters with unsimilarity based on the topological overlap. Three coexpression modules by the dynamic tree-cutting algorithm were identified (**Figure 2C**). The clinical traits provided by the GES54238 database were integrated into the coexpression modules, and those associated with specific clinical traits were identified based on the correlation between the modules and clinical traits, including age, disease condition (normal and advanced HCC), gender, hepatitis B e-antigen (HBeAg), and surface antigen of hepatitis B virus (HBsAg) (**Figure 2D**). Interestingly, the MEturquoise module was positively correlated with the HCC condition representing liver cancer progression from normal liver to advanced HCC (*r*=0.84, *p*=7e-07). The MEturquoise module contained 515 DEGs. Based on weighted value greater than 0.5, we filtered 515 MEturquoise module genes to construct the gene-gene interaction network with 98 nodes and 186 edges (**Figure 2E**).

### Enrichment Analysis of the MEturquoise Module

**Figure 3:**
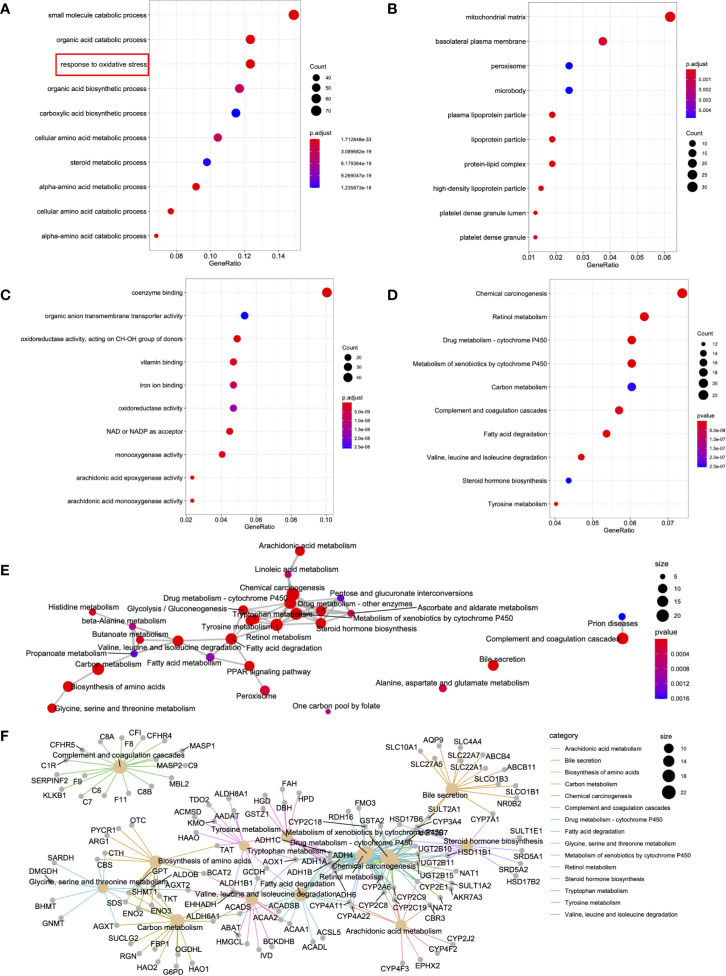
DEG functional enrichment in the MEturquoise module. **(A–D)** The 10 GO terms and pathways with the greatest significance are shown. **(E)** The markedly altered pathways were connected to display the connections. The circle size in the network is directly proportional to the weighted connectivity degree. **(F)** Relationships of those markedly altered DEGs and pathways. The circle size represents the connection number between genes and pathways.

To further understand the biological functions of the 515 DEGs from the MEturquoise module, the R package clusterProfiler function (24) was adopted to carry out functional annotations of the genes. The GO categories were classified as biological processes (BPs), cellular components (CCs), and molecular functions (MFs). In terms of BPs, DEGs were primarily enriched in the “response to oxidative stress,” “organic acid catabolic process,” and “small molecule catabolic process” (**Figure 3A**). Regarding CC, the DEGs were mainly enriched in “basolateral plasma membrane,” “peroxisome,” and “mitochondrial matrix” (**Figure 3B**). Concerning MF, the DEGs were mainly enriched in “organic anion transmembrane transporter activity,” “coenzyme binding,” and “oxidoreductase activity” (**Figure 3C**). The top 10 significantly enriched KEGGs were also presented, including “chemical carcinogenesis,” “drug metabolism,” and “retinol metabolism” (**Figure 3D**). Additionally, network analysis was conducted for the different signal transduction pathways (**Figure 3E**). A portion of certain critical genes related to signal transduction is presented in **Figure 3F**.

### Diagnostic Signature for Prediction in HCC

**Figure 4:**
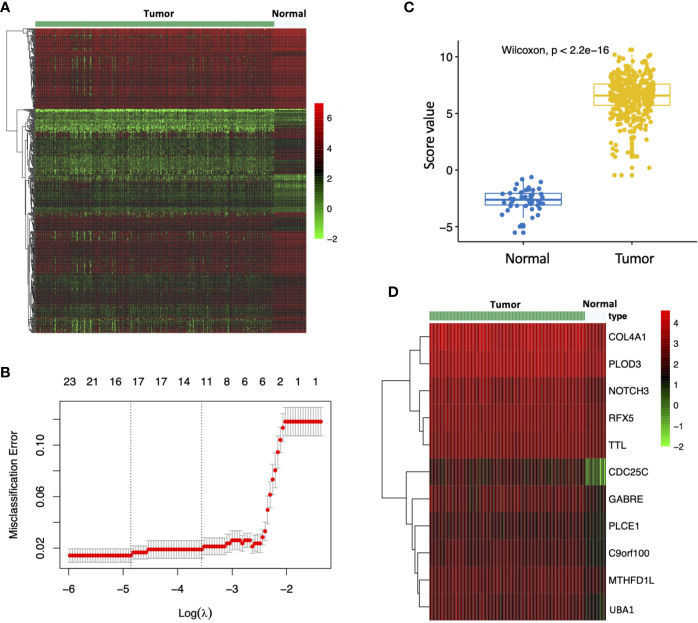
Construction of the diagnostic model. **(A)** Expression pattern of MEturquoise module genes in TCGA HCC RNA-Seq samples (n=423). **(B)** Misclassification error for different numbers of variables revealed by the LASSO regression model. The red dots represent the value of the misclassification error for different numbers of variables in the LASSO regression model, and the gray lines represent the standard error (SE). The vertical dotted lines represent optimal values, indicating the number of the most important genes. **(C)** Distribution of diagnostic score values in different groups. The box plots indicate the median value and interquartile range of diagnostic score values. **(D)** Expression pattern of signature genes identified by the LASSO regression model in TCGA HCC RNA-Seq samples.

To investigate the impact of MEturquoise module on HCC patient diagnosis, we further screened 515 MEturquoise module genes based on 423 TCGA HCC RNA-Seq specimens including 373 HCC samples and 50 nontumor samples (**Figure 4A**). The LASSO regression analysis was applied to construct diagnostic signature. Gene expression profile of the TCGA HCCs and normal controls were applied to the diagnostic model to find key signature genes. After eliminating the overfitting genes by LASSO regression model, 11 candidate genes (*COL4A1, PLOD3, NOTCH3, RFX5, TTL, CDC25C, GABRE, PLCE1, C9orf100, MTHFD1L, UBA1*) were obtained to consist of the diagnostic signature (**Figure 4B**). Then, to evaluate change in expression levels during the hepatocarcinogenesis, we analyzed the evaluation scores for the diagnostic signature of normal controls and HCCs. The evaluation score of individuals with HCC was strikingly upregulated (**Figure 4C**; *p* < 2.2e-16). In addition, the expression pattern of 11 genes for the diagnostic signature in the TCGA HCC cohort is shown in **Figure 4D**.

### Prognostic Signature for Prediction in HCC

**Figure 5:**
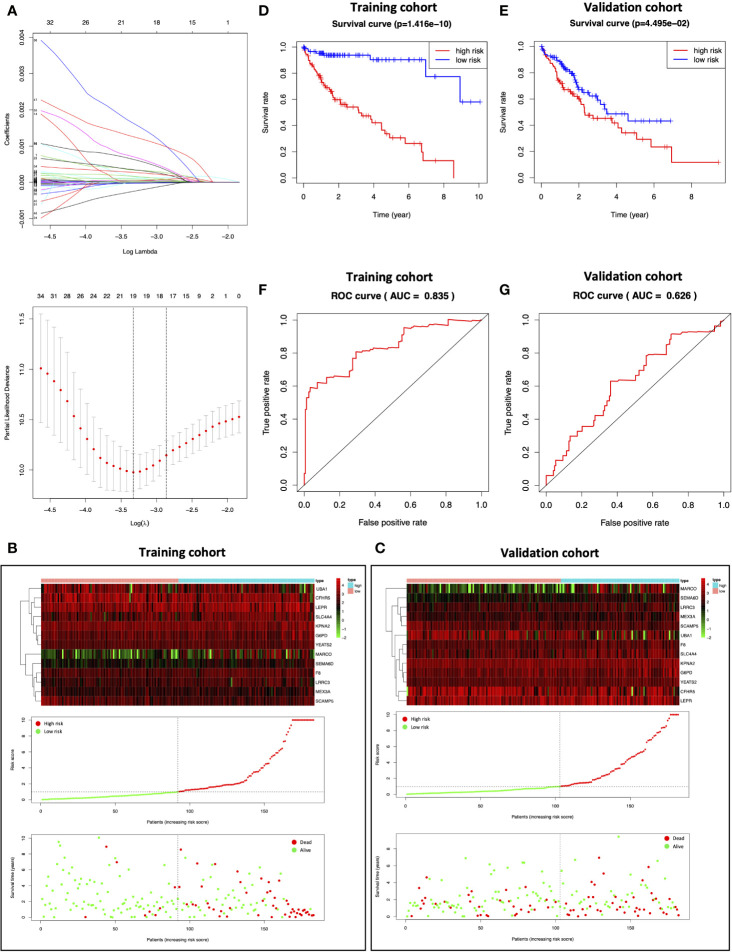
Construction of the prognostic model. **(A)** Determination of the number of factors by LASSO analysis. **(B, C)** The top represents the expression pattern of the prognostic signature genes in the classifiers of the high- and low-risk groups; the center represents the distribution of the risk score; the bottom represents the survival status of patients in the training and validation cohorts. **(D, E)** Kaplan–Meier survival curves of the training and validation cohorts. **(F, G)** ROC curve for the training and validation cohorts.

HCC samples obtained from TCGA were randomized as a training (n=183) or validation (n=182) cohort. To consider the impact of MEturquoise module on HCC patient prognosis, we screened 515 MEturquoise module genes by eliminating the overfitting genes in the LASSO regression algorithm and analyzing by multivariate Cox proportional risk regression (**Figure 5A**); 13 risk genes were thus obtained in the training cohort to establish the prognostic signature, and the risk scores for each sample were analyzed based on the expression levels and regression coefficients of 13 risk genes (24). For the training cohort, the median risk score value was 0.9756, which was adopted as the thresholds to separate samples into the high- or low-risk group (**Figure 5B** center). The distribution of survival status of the training patients was also displayed (**Figure 5B** bottom). The changing trend of the expression level of 13 risk genes in heatmap was concordant with their risk scores in prognostic signature (**Figure 5B** top). While the median risk score in the validation cohort was 0.7653, the capability of the prognostic prediction of risk score was also validated; semblable results were obtained (**Figure 5C**). Based on the Kaplan–Meier survival curves, the high-risk group showed dismal prognostic outcomes compared with the low-risk group in the training cohort (**Figure 5D**; *p*=1.416e-10). Conforming to the results of the training cohort, a poor prognostic outcome was observed in the high-risk group relative to the low-risk group for the validation cohort (**Figure 5E**; *p*=4.495e-02). Additionally, receiver operating characteristic (ROC) curves were plotted, and the area under the curve (AUC) values (0.835 and 0.626, respectively) were high for both cohorts (**Figures 5F, G**).

### Prioritization of Potential Novel Markers

**Figure 6:**
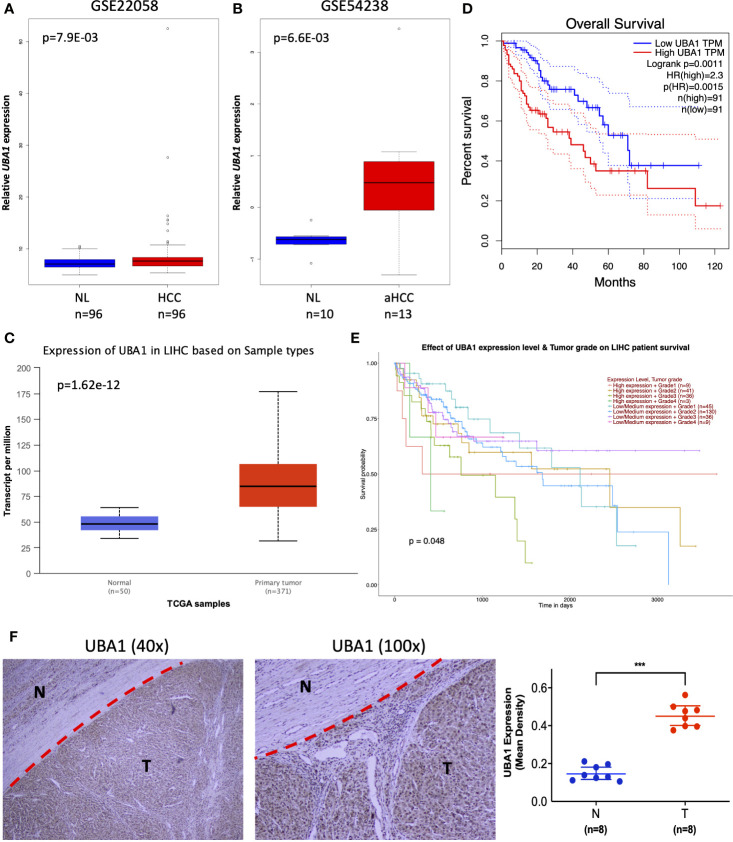
Clinical implication of *UBA1*. **(A–C)** The *UBA1* expression levels are shown in GSE22058 (n=192), GSE54238 (n=23), and TCGA HCC data (n=421). **(D)** Kaplan–Meier survival curve of differentially expressed *UBA1* (HR=1.49; *p*=0.026). The red dotted line above the red curve and red dotted line below represent the high (50%) and low cutoff (50%) on the survival curve. The blue dotted line above the blue curve and blue dotted line below represent high (50%) and low cutoffs (50%) on the survival curve. **(E)** Differentially expressed *UBA1* is associated with HCC grade (*p*=0.048). **(F)** Representative images (left) and quantification (right) of *UBA1* immunohistochemical staining in eight HCC and paired nontumor tissues, ***p < 0.001.

To further mine the most valuable genes in the above signature genes, the clinical significance of the signature genes was analyzed. A preliminary screening showed that the ubiquitin-like modifier activating enzyme 1 (*UBA1*) gene had remarkable clinical value and was identified in both diagnostic and prognostic signature genes. *UBA1* exhibited high expression in HCC samples in the GSE22058 (n=192), GSE54238 (n=23), and TCGA HCC RNA-Seq dataset s (n=421) (**Figures 6A–C**), and the increasing expression of *UBA1* was significantly associated with poor survival in the Cancer Genome Atlas Liver Hepatocellular Carcinoma (TCGA-LIHC) cohort (**Figure 6D**; n=369; HR=1.49; *p*=0.026). Additionally, the differential expression of *UBA1* was strikingly correlated with tumor grade (*p*=0.048) (**Figure 6E**). Next, we examined *UBA1* expression in eight paired clinical HCC and nontumor tissue samples. The immunohistochemical (IHC) assay revealed that *UBA1* protein expression levels were remarkably higher in HCC than in the corresponding nontumor tissues (**Figures 6F**). Therefore, these results indicated that the upregulation of *UBA1* might be a promising marker for the diagnostic and prognostic prediction of liver cancer.

**Figure 7:**
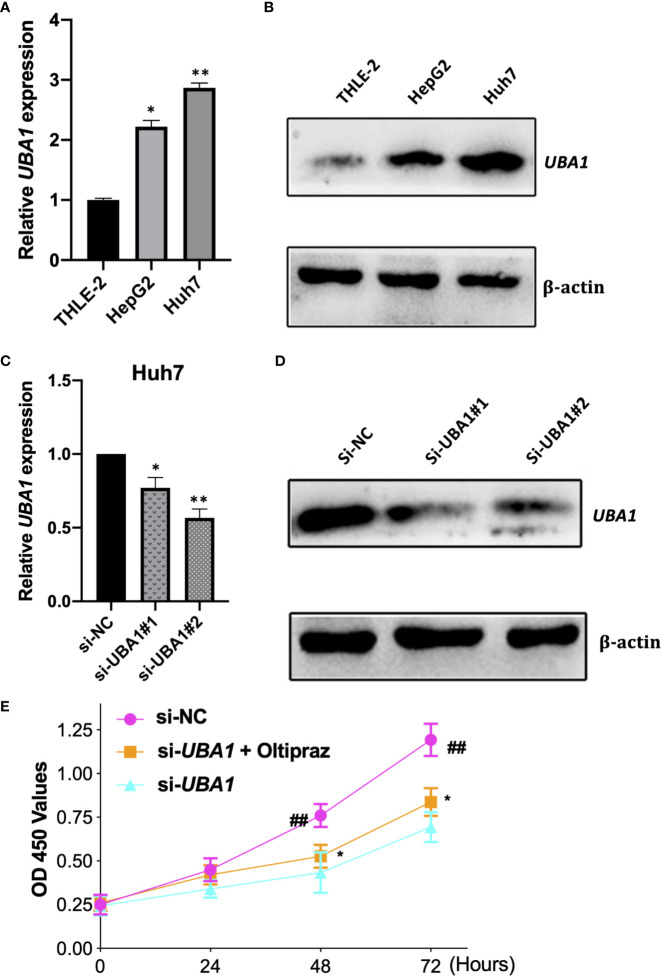
Verification of *UBA1* levels in HCC cells. **(A, B)** *UBA1* expression in some HCC cell lines was detected by qRT–PCR and Western blotting. **(C, D)** Effects of *UBA1* interference within Huh7 cells at the mRNA or protein level. **(E)** Cell proliferation ability detected by CCK-8 viability following *UBA1* expression interference. (*p < 0.05; **p < 0.01; ^##^*p* < 0.01).

THLE-2, HepG2, and Huh7 cells were later used to extract total RNA and proteins to perform qRT–PCR and Western blotting assays, respectively. The *UBA1* expression levels were increased in HepG2 and Huh7 cells by qRT–PCR and Western blotting analysis compared with immortalized noncarcinoma human liver cells (THLE-2) (**Figures 7A, B**). According to our findings, *UBA1* might play a vital role during liver cancer development. Notably, the *UBA1* level was higher in Huh7 cells than in HepG2 cells. Therefore, Huh7 cells were used in subsequent cell analyses.

### *UBA1* Silencing Inhibits the Malignant Phenotypes and Ferroptosis of Liver Cancer Cells

**Figure 8:**
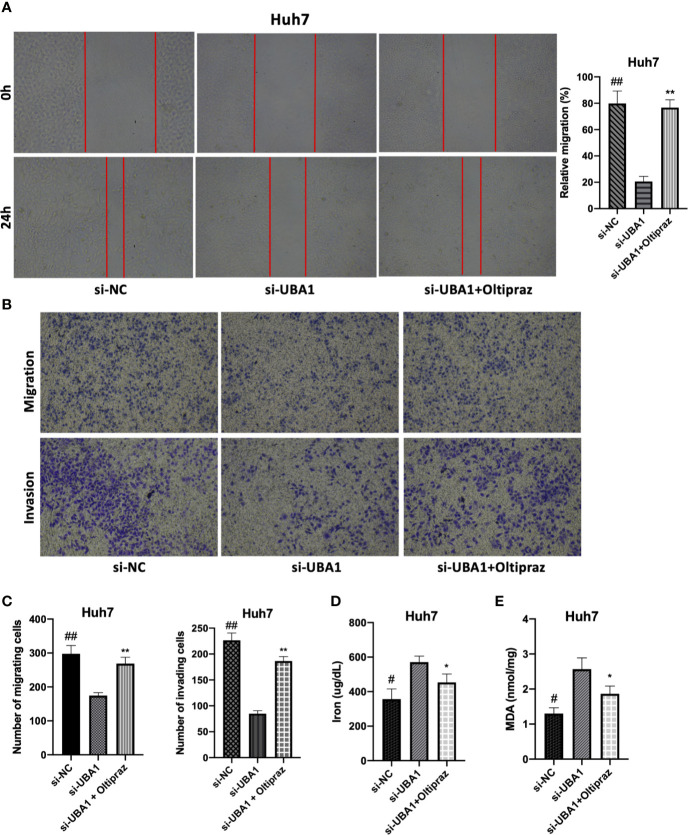
Effects of changed *UBA1* expression on malignant phenotypes and ferroptosis in HCC cells. **(A)** Wound healing assay. Migratory cells to the wound were determined following different treatments and imaged. **(B, C)** Transwell migration and invasion assays. Huh7 cells treated with different drug doses were analyzed for their invasion and migration capacities. **(D)** Ferrous iron and the MDA content **(E)** in Huh7 cells subjected to si-*UBA1* transfection (* si-*UBA1* versus si-*UBA1*+oltipraz, **p < 0.01, *p < 0.05; # si-NC versus si-*UBA1*, ^##^*p* < 0.01, ^#^*p* < 0.05).

To elucidate the biological function of *UBA1* in the progression of HCC, Huh7 cells were subjected to lentivirus-mediated *UBA1* shRNA (shRNA #1 and #2) transfection. The *UBA1* mRNA expression of Huh7 cells transfected with sh-*UBA1*#1 and #2 was significantly downregulated, suggesting successful *UBA1* knockdown (**Figures 7C, D**). sh-*UBA1* showed better activity and was then screened in subsequent assays. Compared with the NC-siRNA group, the *UBA1*-shRNA group displayed remarkably reduced Huh7 cell proliferation (**Figure 7E**). Additionally, *UBA1* knockdown cells showed reduced migratory or invasive abilities relative to those of the NC-siRNA group (**Figures 8A–C**). Thus, *UBA1* was necessary for Huh7 cell migration and invasion.

Afterwards, ferroptosis-associated indices, such as the accumulation of iron and lipid peroxidation following *UBA1* interference, were assessed to determine the relationship between *UBA1* and ferroptosis in Huh7 cells. Thus, *UBA1* is essential for the changed Fe^2+^ levels because ferrous iron (Fe^2+^) was responsible for initiating ferroptosis. *UBA1* knockdown upregulated the Fe^2+^ content in cells (**Figure 8D**). Moreover, MDA, the typical lipid peroxidation end-product, was tested in Huh7 cells for its accumulation regulated by *UBA1*. *UBA1* suppression promoted MDA accumulation within Huh7 cells (**Figure 8E**). These results indicated that *UBA1* might serve as a ferroptosis regulator that regulates the MDA and Fe^2+^ levels within Huh7 cells.

### *UBA1* Silencing Suppresses the *Nrf2* Signal Transduction Pathway

**Figure 9:**
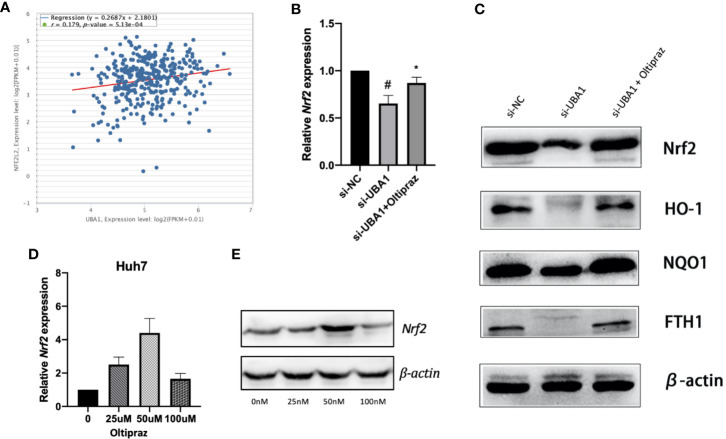
The *Nrf2* signal transduction pathway. **(A)** Coexpression correlation analysis in the TCGA HCC RNA-Seq cohort. **(B)** Following *UBA1* silencing, *Nrf2* expression, along with the downstream members (*HO-1*, *NQO1*, and *FTH1*) **(C)**, was downregulated, as evidenced by Western blotting. **(D, E)** Best oltipraz dose screening. *p < 0.01, ^#^*p* < 0.05.

As illustrated by enrichment analysis (**Figure 3A**), OS might play a vital role during HCC progression. Correlation analysis was performed for HCC samples obtained from the TCGA HCC RNA-Seq cohort to examine the association between *UBA1* and the key OS gene *Nrf2*. The *UBA1* level was positively correlated with *Nrf2* (**Figure 9A**; r=0.179, *p*=5.13e-04) and might serve as the key gene involved in the OS response pathway. Furthermore, *UBA1* knockdown significantly downregulated *Nrf2* levels (**Figure 9B**), as well as the expression of downstream genes (*HO-1*, *NQO1*, and *FTH1*) (**Figure 9C**). Furthermore, oltipraz, an *Nrf2* activator, was used to treat cells at different levels to determine the relationship between *Nrf2* pathway activation and the changed inhibition of *UBA1* knockdown in Huh7 cells. Huh7 cells were then treated with 50 µM oltipraz following *UBA1* knockdown (**Figures 9D, E**). Thus, combined oltipraz treatment completely reversed the inhibition of *UBA1* knockdown on cell proliferation, invasion, metastasis, and ferroptosis (**Figures 8A–G**). Regarding *Nrf2* pathway members (*HO-1*, *Nrf2*, *FTH1*, and *NQO1*), their expression increased under oltipraz. This result intuitively suggests the effect of *UBA1* on regulating HCC cell ferroptosis and biological behaviors through the *Nrf2* pathway.

## Discussion

In this study, expression patterns were acquired based on the GSE22058 and GSE54238 datasets, and 926 DEGs were found to be shared by these two datasets, which were used in systemic bioinformatic analysis. *UBA1*, a new possible gene, was selected to explore its crucial role in HCC cell proliferation, invasion, migration, and ferroptosis, together with the possible related mechanism. To our knowledge, the present work offers sound evidence to illustrate the role of *UBA1* in modulating ferroptosis in HCC cells through the *Nrf2* pathway.

Ferroptosis represents a new type of cell death that is iron dependent. In recent reports, the effect of ferroptosis is affected by diverse biological backgrounds of cancers. The Nrf2-Keap1 signaling pathway decreases ferroptosis by switching the malignant proliferation of non-small cell lung cancer cells (25). The Sigma-1 receptor (S1R) limits ferroptosis by hindering the expression of glutathione peroxidase 4 in HCC cells (26). However, the ferroptosis mechanisms for cancer cells remain incompletely understood.

Recent studies have demonstrated that ubiquitin-like modifier activating enzymes have essential effects in human tumors. For example, ubiquitin-like modifier activating enzyme 2 (*UBA2*) induces the migration and invasion of gastric cancer cells (27). *UBA7* serves as a potential biomarker for diagnostic and prognostic prediction in breast cancer (28). *UBA1*, ubiquitin-like modifier activating enzyme 1, is required for the cellular response to DNA damage (29). *UBA1* was reported to be associated with several cancers (30). Blocking *UBA1* decreased the levels of ubiquitylated proteins, increasing markers of proteotoxic stress and DNA damage stress in acute myeloid leukemia (31). Furthermore, silencing *UBA1* reduced the abundance of ubiquitinated proteins in leukemia and myeloma cells and promoted cell death, and in mouse models of leukemia and multiple myeloma, inhibiting *UBA1* expression strikingly diminished tumor weight and volume (32). However, the biological effects of *UBA1* are less well understood in HCC. In our studies, we screened *UBA1* from the signature genes of diagnostic and prognostic models and determined that its expression levels were strikingly upregulated in recruiting HCC tissues and cell lines. To interpret the clinical implication of *UBA1* in HCC, we showed that high *UAB1 expression* was strongly associated with poor survival in HCC patients, and its differential expression was related to tumor grade. The silencing of *UBA1* has been shown to repress the proliferation, migration, invasion, and increase ferrous iron and MDA levels in live cancer cells. Thus, *UBA1* is an independent indicator for HCC progression.

*Nrf2* was found in prior research to be an important OS-related gene during tumorigenesis (33, 34). *Nrf2* silencing was found to inhibit HCC progression (35, 36), implying the carcinogenic role of *Nrf2* during HCC development by achieving downstream targets. As found in this study, *UBA1* was positively correlated with *Nrf2*, which regulates the ferroptosis and malignant phenotypes of HCC cells *via* the *Nrf2* pathway; these effects were reversed by oltipraz, the *Nrf2* activator. Nuclear factor erythroid-2-related factor 2 (*Nrf2*) mainly modulates various genes containing antioxidant response elements (AREs) in promoter regions (37). *Nrf2* activation occurs in the context of HCC because *UBA1* upregulation initiates AREs to hinder ferroptosis and promote HCC cell proliferation, invasion, and migration. The role of Nrf2 in *UBA1 in vivo* should be determined in future studies.

To our best knowledge, this study is the first to demonstrate that *UBA1* promotes HCC cell proliferation, invasion, and migration but suppresses ferroptosis *in vitro*, elucidating that *UBA1* plays a vital role in HCC development. *Nrf2* was previously identified as a promising therapeutic target for HCC (38, 39). Additionally, targeting abnormal *UBA1* expression may serve as a well-tolerated treatment. Based on this study, the suppression of *UBA1*, an *Nrf2* regulator, may serve as a candidate treatment strategy for HCC. However, this treatment should be validated *in vivo* and further developed to treat HCC.

## Conclusions

In summary, the results in this study suggest that DEGs are found based on two different HCC datasets, and then the upregulated *UBA1* gene was screened among those genes for diagnosis and prognosis prediction. *UBA1* participates in HCC cell ferroptosis through the *Nrf2* pathway. This work offers an important avenue to unveil the new mechanisms of HCC development.

## Data Availability Statement

Publicly available datasets were analyzed in this study. This data can be found here: the NCBI Gene Expression Omnibus (GSE22058 and GSE54238).

## Ethics Statement

The studies involving human participants were reviewed and approved by The Research Ethics Committee of Sichuan Cancer Hospital. The patients/participants provided their written informed consent to participate in this study.

## Author Contributions

All authors contributed to the article and approved the submitted version.

## Funding

This work was sponsored by National Natural Science Foundation of China (Grant nos. 81904218 to QP and 82074437), China Postdoctoral Science Found (2019M653831XB to QP), and Natural Science Foundation of Chongqing, China (cstc2019jcyj-msxmX0095 to QP).

## Conflict of Interest

The authors declare that the research was conducted in the absence of any commercial or financial relationships that could be construed as a potential conflict of interest.
